# Does fixed-angle plate osteosynthesis solve the problems of a fractured proximal humerus? A prospective series of 87 patients

**DOI:** 10.1080/17453670902807417

**Published:** 2009-02-01

**Authors:** Peter Helwig, Christian Bahrs, Björn Epple, Justus Oehm, Christoph Eingartner, Kuno Weise

**Affiliations:** ^1^Department of Orthopaedics and Traumatology, Albert Ludwigs UniversityFreiburgGermany; ^2^Department of Trauma and Reconstructive Surgery, BG Trauma CenterTübingenGermany; ^3^Department of Trauma and Reconstructive Surgery Unit, Caritas Hospital, Bad MergentheimGermany

## Abstract

**Background and purpose** There is considerable controversy about the treatment of complex, displaced proximal humeral fractures. Various types of head-preserving osteosynthesis have been suggested. This prospective case series was designed to evaluate the perioperative and early postoperative complications associated with fixed-angle implants and to record outcome after bone healing.

**Patients and methods** Fractures of the proximal humerus were stabilized surgically in 87 patients (mean age 64 (16–93) years) by application of a fixed-angle plate (65 PHILOS, 22 T-LCP). There were 34 2-segment fractures, 42 3-segment fractures, and 11 4-segment fractures, including 7 dislocation fractures. Follow-up assessment after a minimum of 12 months was based on the Constant, UCLA, and DASH scores and on radiographs.

**Results** Postoperative complications included soft tissue problems (n = 9), humeral head necrosis (n = 9), screw perforation (n = 11), secondary displacements (n = 14), and delayed fracture healing (n = 4). Treatment outcomes recorded on the various scores were very good in 60–82% of the cases.

**Interpretation** Screw perforation of fixed-angle implants has replaced the complications of secondary displacement and implant loosening after using conventional plates. Even with the use of fixed-angle implants, fractures of the proximal humerus are associated with a high complication rate and sometimes poor outcome.

## Introduction

Proximal humerus fractures are increasingly common in the elderly (Bengner et al. 1988). The fractures are differentiated according to various classifications that take account of displacement and number of fragments (Duparc and Largier 1976, Jakob et al. 1984, Edelson et al. 2004, Neer 2006). Treatment methods range from nonoperative to surgical head-preserving stabilization and total joint replacement depending on the extent of displacement and fragmentation. There are various surgical head-preserving methods with different kinds of plates, external fixation (Martin et al. 2006), intramedullary devices (Sosef et al. 2007) and K-wire procedures (Resch et al. 2001), sometimes combined with the use of anchoring devices.

The most frequent injuries are undisplaced 2-segment fractures as classified according to Neer (Lind et al. 1989), for which it is generally accepted that nonoperative management will lead to good outcomes (Koval et al. 1997). There is no consensus about the management of displaced 3- and 4-segment fractures (Zyto 1998, Doursounian et al. 2000, Hintermann et al. 2000, Resch et al. 2001, Hente et al. 2004, Meier et al. 2006). Poor outcomes are common in these types of fractures, and may be due to reduced humeral head blood supply and difficulties in achieving and maintaining exact fracture reduction with an appropriate stabilization method. In addition, secondary loss of reduction frequently occurs (Hessmann et al. 1999). Likewise, joint replacement procedures have also led to disappointing results (Zyto et al. 1998, Mehlhorn et al. 2006).

Our prospective case series focuses on perioperative and early postoperative complications in proximal humerus fractures treated with fixed-angle implants. We also assessed whether outcome depends on classification of the fracture or on the quality of the immediate result of postoperative fracture reduction.

## Patients and methods

After approval by the local ethics committee (June 27, 2002 and September 30, 2004; reg. no. 347/2004V) we prospectively included 87 proximal humeral fractures in this study. All fractures were stabilized surgically with a fixed-angle plate from November 2002 through December 2004. Approximately 100 proximal humerus fractures are treated annually in the Department of Trauma and Reconstructive Surgery of the BG Trauma Center, Tübingen (a level-1 trauma center).

We included patients with displaced, unstable proximal fractures of the humerus, provided the humeral head was not stripped of soft-tissue attachments and was technically reconstructable. Patients were included independently of the fracture type if they had a mature skeleton and if there had been a delay between accident and surgery of not more than 10 days.

Patients with pseudarthrosis, pathological fractures and refractures, open fractures, or concomitant fractures of the ipsilateral distal humerus or elbow were not included. Also excluded were patients with disorders affecting the healing process—such as multiple sclerosis, paraplegia, or pre-existing plexus injury—and polytraumatized patients with an injury severity score (ISS) of greater than 16.

36 men and 51 women with a median age of 64 (16–93) years were included. There were 31 cases of high-energy trauma and 56 cases of low-energy trauma (domestic fall or fall from a standing position). Standard radiographs in two planes (AP and axial) were obtained for all patients and were used to plan the surgical procedure. CT-scan was reserved for special cases. In rare cases, stability or instability was diagnosed by dynamic fluoroscopy to identify the indication for surgery.

Fractures were categorized with reference to the AO and Neer classifications. According to the AO classification, there were 35 type A, 39 type B, and 13 type C fractures. In the Neer classification, there were 34 2-segment fractures, 42 3-segment fractures, and 11 4-segment fractures and 7 dislocation fractures (four 4-segment fractures and three 3-segment fractures; median age of patients: 39 (33–75) years). All procedures were performed by or assisted by experienced trauma surgeons with at least 10 years of training. The implanted devices were 87 fixed-angle implants including 22 fixed-angle 4.5-mm L- and T-plates (Stratec Medical, Oberdorf, Switzerland) and 65 Philos plates (Synthes GmbH, Solothurn, Switzerland).

If necessary, surgery took place within 1 week of the accident as an emergency procedure (dislocation fractures, severe fracture displacement). The patient was placed in beach-chair position on a radiolucent table, with side placement of an image intensifier that would allow viewing of the humeral head in two planes. The approach was either an anterior deltoid split or through the delto-pectoral groove. After an initial learning curve, surgical technique for the Philos plate was modified by changeover to an anterior deltoid split approach without application of the aiming device. Extensive exposure of the fracture was avoided (Hessmann et al. 1999). Fracture reduction was achieved through indirect manoeuvres and/or with the help of an elevatorium or K-wires used as joysticks for reduction of the shaft-head displacement, and with sharp bone hooks for reduction of the tuberosities. The plate was placed at least 5–8 mm distal to the upper end of the greater tuberosity and 2–4 mm lateral to the bicipital groove.

The fracture reduction achieved was temporarily fixed with K-wires to facilitate image intensifier control, whereupon the wires were inserted through the holes of the Philos plates. When anatomical reduction was obtained, insertion of the screws was performed. An intraoperative image intensifier was used to control the angular stable screw position in the humeral head in true AP and transaxial view to avoid intraarticular screw positioning. Additional screws were implanted in 2 patients, and 1 patient required additional tension band wiring.

Postoperatively, the shoulder was immobilized in a sling for 2–7 days followed by active movement up to 90° abduction and free flexion and retroversion for 4 weeks after surgery; then free, active mobilization was allowed. Implant removal after healing of the fracture was suggested to younger patients (< 60 years).

The DASH score was recorded for all patients preoperatively. All patients had clinical and radiographic follow-up for at least 1 year postoperatively, with a median follow-up time of 27 (12–73) months. At follow-up the DASH, Constant, and UCLA scores were recorded separately for each shoulder (Table).

**Table:** Shoulder scores at follow-up

Score:	Constant-Murley (max. 100 points)	UCLA (max. 35 points)	DASH (max. 0 points)
Very good	86–100	34–35	0–20
Good	71–85	28–33	21–40
Satisfactory	56–70	21–27	41–60
Poor	< 55	< 20	> 61

Perioperative and early postoperative complications such as hematoma and infection were recorded. Residual axial and rotational deformities of the head fragment and/or greater tuberosity immediately after surgery were assessed radiographically according to Bahrs et al. (2007) as follows. A score of zero is obtained if a, b, and c are met: (a) greater tuberosity below the level of the cortex or a side-to-side difference of < 5 mm; (b) no increased varus or valgus (±15°) of the head fragment in the AP view; (c) no increased retro- or antetorsion (±15°) of the head fragment in the axial projection. If 2 of the criteria are met, the score is 1; a score of 2 is achieved if one of the criteria is met.

Secondary displacement of the fracture, screw perforation (cut-out), humeral head necrosis classified according to Cruess (1976), plate impingement, and delayed fracture healing/pseudarthrosis were also recorded.

All radiographs were analyzed by 3 independent investigators.

### Statistics

Univariate regression analysis (ANOVA) with JMP 6 software (SAS Institute Inc., Cary, NC) was used to assess which factors influenced the final score. The level of significance was set at p ≤ 0.05.

## Results

**Figure 1.**
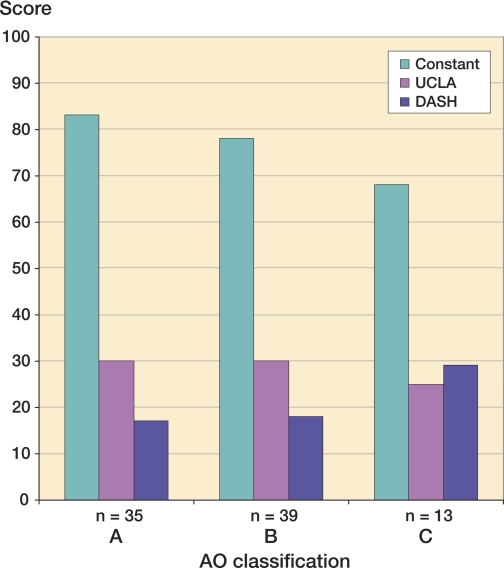
Fracture distribution (AO classification) and outcome scores.

**Figure 2.**
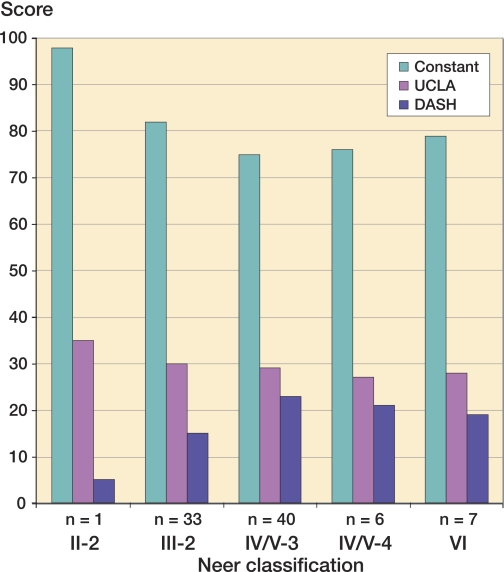
Fracture distribution (Neer classification) and outcome scores.

At follow-up, an outcome ranging from good to very good was found in 52 and 71 cases (UCLA and DASH scores, respectively) (Table). A borderline correlation was found between UCLA score and the age of the patient (p = 0.04). The UCLA score correlated with the AO classification (divided into types A, B and C; p = 0.02). The Constant-Murley and DASH scores showed no significant association with the Neer (p = 0.3) or the AO classifications (p = 0.1) (Figures 1 and 2).

There were no complications during the immediate postoperative course in 78 patients. In 9 cases treated with Philos plates, a superficial postoperative infection or hematoma (4 cases) requiring revision surgery occurred. No correlation was found between treatment outcomes according to the various scores and the need for soft tissue revision (p = 0.7–1.0).

Radiographic alignment of the anatomy immediately after surgery was achieved in 69 cases. The restoration of the anatomy was better for 2-segment fractures than for complex fractures (p = 0.01). The final Constant score and the final UCLA score were influenced by correct alignment of the fracture (p = 0.001 and p < 0.001, respectively).

Screw perforation through the humeral head (so-called cut-out) was observed in 11 patients. These included two 2-segment fractures, seven 3-segment fractures, and two 4-segment fractures. 8 of the 11 patients with cut-out achieved only a poor to satisfactory outcome score. This complication was seen more frequently for fractures that could not be anatomically reduced (p < 0.001). In all fractures with considerable axial or rotational deformities and/or non-anatomically reduced greater tuberosity, subsequent cut-out was evident in 8 cases. Regardless the fracture type (according to the Neer and AO classifications), secondary displacement occurred in 14 cases. Secondary fracture displacement was frequently recorded for non-anatomically reduced fractures (p < 0.001).

Within the first postoperative year, 6 cases of partial (grade II) and 9 cases of complete (grade III) humeral head necrosis were found (8 times after 3-segment fracture, 5 times after 4-segment fracture, and twice after 2-segment fracture), but no joint replacement was necessary in any patient. A correlation was found between manifestation of humeral head necrosis and residual axial or rotational deformities (p = 0.004) and secondary fracture displacement (p = 0.03).

There was no correlation between the presence of humeral head necrosis and the number of fragments classified according to Neer (p = 0.07) or the patient’s age (p = 0.9).

The implants were removed in 38 patients, in 12 cases due to plate-related complications such as cut-out or plate impingement. One case of pseudarthrosis and 3 cases of delayed union were observed. No surgical revision was performed in these cases, due to the fact that the patients did not give their consent.

## Discussion

All classification systems are subject to inter- and intraobserver variability (Siebenrock and Gerber, 1992). In view of the popularity of the Neer and AO systems, we classified the fractures in our study according to these despite their inevitable limitations. The similarity in frequency of 3- and 4-segment fractures in this study to the results of other working groups (Kettler et al. 2006, Bahrs et al. 2007) allows us to make comparisons. The unusually high rate of revision surgery for early postoperative hematoma and infection associated with the Philos plates must be attributed to the initial learning curve for the system, since no hematoma or infection occurred after modification of the surgical technique without application of the aiming block provided by the manufacturer. If the screws are inserted with the help of the aiming block, the soft tissues must be retracted using wound spreaders for a longer period of time. Hepp et al. (2008) found an influence of the surgical approach on the final outcome, something that we did not investigate in this study.

The high number of plate-related surgical revisions can be attributed almost exclusively to cut-out—which, nevertheless, was found to occur far less frequently than the poor results achieved with the cannulated, angled blade plate (Meier et al. 2006). Penetration of the screw through the previously intact joint surface has been identified as a frequent complication by all working groups who have evaluated outcomes after fixed-angle plate osteosynthesis (Kettler et al. 2006, Voigt et al. 2007). It has taken the place of plate and screw loosening as the main implant-related complication of non fixed-angle implants. We found an important statistical association between intraoperative anatomical reconstruction and cut-out and/or secondary displacement. These results have also been confirmed by Gardner et al. (2007), who specified the medial buttress as the key criterion of anatomical reconstruction.

According to Dimakopoulos et al. (2007), it is possible to treat almost all proximal humeral 2-, 3-, and valgus impacted four-part fractures with sutures only. Perhaps the combination of sutures and fixed-angle plate might improve the results, especially in osteoporotic bone. Thus, we now recommend additional sutures to reduce and fix the tuberosities.

Surprisingly, we found similar outcome for the different implants in relation to plate-related problems. The Philos plate is of flat design, which enables it to be implanted very proximally and lateral to the bicipital groove without causing impingement. We expected a higher rate of subacromial impingement-related problems for the T-LCP because of the lack of anatomical configuration of this plate. However, in contrast to results recorded for similarly contoured plates—such as the clover-leaf plate (Kuchle et al. 2006) or the conventional 4.5-mm T-plate (Bahrs et al. 2007)—we did not observe any implant-related impingement caused by the T-LCP.

The subjective and objective treatment outcomes were not dependent on the Neer classification, but sometimes on the AO classification. No correlation between the final score outcome and the Neer classification has been found in other studies (Kettler et al. 2006, Bahrs et al. 2007). We found a significant relationship between a poor score and a poor anatomical reconstruction. We also found a negative influence of secondary fracture displacement on the final score. Thus, special emphasis on anatomical fracture-reconstruction is important and appears to be the most important aspect of surgical stabilization with angular-stable implants. Furthermore, accurate intraoperative image intensifier viewing is mandatory—and it may help to prevent intraarticular placement of screws or drilling through the intact articular surface, which may precede the later cut-out.

The results of the 7 dislocation fractures (four 4-part fractures and three 3-part fractures) were not significantly different from those of the 3- and 4-part fractures. This is consistent with the investigations of Bastian and Hertel (2008), where an initial humeral head ischemia was not found to be predictive of humeral head necrosis later on if anatomical reconstruction was achieved and fixed. In their investigation of 58 dislocation fractures, Robinson et al. (2006) concluded that these fractures must be subclassified further into type I and II fractures. All of the type I fractures could be stabilized with good results, and type II fractures could be stabilized in younger patients (< 60 years). Similar results were reported by Gerber et al. (2004) with only 2 cases of humeral head necrosis in 33 articular fractures, with 8 dislocation fractures requiring hemiarthroplasty later on. Similar to our results, Trupka et al. (1997) found that for 3- and 4-part fractures (with and without dislocation fractures), there were comparable Constant scores in both groups.

Only 60–82% of our patients with a proximal humeral fracture that was stabilized with an angular-stable plate achieved good to very good results using the various scores. We conclude that despite the use of fixed-angle implants, fractures of the proximal humerus still show a high complication rate and sometimes only an acceptable outcome. Fixed-angle implants did not improve the results of surgery for complex proximal humeral fractures.
